# General Pathology of Eruptive Fevers, and the Principles That Should Govern Their Treatment

**Published:** 1862-10

**Authors:** N. S. Davis

**Affiliations:** Professor of Clinical Medicine, &c.


ARTICLE XXXIV.

### GENERAL PATHOLOGY OF ERUPTIVE FEVERS, AND THE PRINCIPLES THAT SHOULD GOVERN THEIR TREATMENT.

Read to the Chicago Medical Society, Nov. 7, 1862.

By N. S. DAVIS, M.D., Professor of Clinical Medicine, &c.

The diseases directly alluded to, under the head of eruptive fevers, in this paper, are Variola, Rubeola, and Scarlatina. Their great relative importance is shown by the fact, that in England and this country, and probably throughout all the civilized countries, from *eight* to *ten* per cent of the entire mortality arises from these three diseases.

A glance at the statistics of mortality show another interesting fact, namely, that while one of these diseases is most prevalent and fatal one year, and another the next year, the annual aggregate mortality from them all remains nearly uniform. It also appears from the same statistical tables, that while the mortality from small-pox has been greatly diminished by the general practice of vaccination during the last half century, the mortality from scarlatina, rubeola, and hooping-cough has correspondingly increased, thereby maintaining the annual aggregate nearly the same. This has led Dr. Gregory, and some others, to claim that there exists among the class of acute eruptive fevers a "law of compensation," or vicarious prevalence.

Aside from the intimate relation which the several eruptive fevers bear to each other, as shown by the statistics already alluded to, there are several interesting characteristics common to them all:—

1st. While they often occur epidemically they are all propagable by contagion.

2d. There is in the human species an almost universal susceptibility to these fevers. The most liberal estimates do not claim, as exempt from susceptibility, more than one in sixty of the entire population.

3d. While there is an almost universal susceptibility to each of the diseases under consideration, such susceptibility is permanently destroyed by a single attack. Hence it is very rare that the same individual is attacked twice by the same eruptive fever.

4th. Each of these diseases are not only self-limited in duration, but they present several well-defined stages or epochs: as the period of incubation, the promonitory fever, and the period of eruption.

These several characteristics plainly indicate the fact, that this class of diseases arise from exciting causes of a specific and uniform character; causes which are generally ranked as animal poisons, and capable of being reproduced in the body of the sick. They may be introduced into the system either by contact with the cutaneous surface, or by inhalation and absorption from the pulmonary mucous membrane.

When introduced into the blood by either of these methods, even in extremely minute quantity, no immediate appreciable effect is produced; but in a period varyiug from four to fourteen days, it becomes multiplied in the blood to such an extent as to disturb the susceptibility of all the organized structures of the body, thereby causing all the symptoms of an active irritative grade of general fever. That these poisons do thus effect the blood, and through it the properties of all the tissues, is demonstrated by the fact that inoculation with the blood of patients laboring under the active stage of rubeola and scarlatina, communicates the same disease to the persons inoculated. By what process these poisons are multiplied in the blood during the period of incubation is not known. From an early period, it has been regarded as analagous to fermentation in vegetable matter. This idea was distinctly inculcated more that two hundred years ago, by SYDENHAM and DIEMERBRŒCK. In modern times, it has been revived and rendered somewhat popular by LIEBIG.

From the supposed analogy to the process of fermentation, the process of incubation in the eruptive fevers has been called *zymosis*, from the Greek word signifying *ferment;* and they have been styled *zymotic* diseases, by a large class of modern writers.

Whether there is any reality in the analogy here alluded to or not, it is quite certain that by some process the smallest quantity of variolous, rubeolous, or scarlatinous poison introduced into the blood undergoes such rapid and extensive multiplication as to induce, in a few days, a high grade of febrile excitement throughout the whole system. At the same time, it effects such a change, either in the composition of the blood or the susceptibility of the tissues, or both, as to destroy all influence of the same poison at any future period. In addition to the action of eruptive fever poisons on the susceptibility of the tissues generally, they all display a special affinity for the cutaneous structures, in which their presence induces more or less inflammation constituting the eruption. The affinity of those poisons which gives rise to the pustular form of eruption, as the variolous, is so strong as to cause their lodgement in the skin during the first four days of the febrile action, and their consequent removal from the mass of the circulating fluids. This causes simultaneously a subsidence of the general irritative febrile action, and the development of a local pustular inflammation at each point in the skin where the poison has been deposited. If all the poison is thus early removed from the blood, on the appearance of the eruption, the apyrexia becomes complete, and the patient remains free from fever until the intensity of the cutaneous inflammation renews more or less general febrile action. But if, as sometimes happens, some of the poison fails to be deposited in the skin and continues in the blood, the subsidence of the fever will not be complete on the appearance of the eruption, but will continue with more or less severity, accompanied by progressive deterioration of the blood, and all those symptoms set forth by authors as characterizing the malignant form of variola.

The poisons producing the eruptive fevers of the exanthematous variety, such as rubeola and scarlatina, display a similar affinity for the skin, and in addition also for the mucous membrane of the fauces and larger bronchial tubes; exciting in these structures more or less inflammation of a specific character, but do not become deposited in these tissues to such an extent as to free the blood from their presence, or to relieve the other structures of the body from their irritating effects. Consequently, there is, in these diseases, no abatement of the febrile symptoms on the appearance of the rash or local imflammation in the skin. On the contrary, the fever continues in full severity until the eruption itself begins to decline. That the specific poison continues in the blood during the eruptive as well as premonitory stage, would seem to be demonstrated by the fact that these diseases can be communicated to healthy persons by inoculation with the *blood* of the sick. That it is finally expelled partly through the skin and partly through the kidneys, we have no doubt. That the latter organs take part in the process of expulsion of the poison, and consequently suffer much derangement of their function thereby, is countenanced by the fact that in a large proportion of cases, especially of scarlatina, the urine becomes albuminous during the progress of the disease. And dropsical effusions, produced by deficient and unnatural renal secretion, are well known to be among the most frequent sequalæ of these fevers.

With these explanations, we may sum up our views of the pathology of eruptive fevers as follows:—1st. There is an exaltation of the elementary susceptibility of the organized tissues of the body, with a perversion of vital affinity, as indicated by an active grade of irritative fever and disturbance of the great functions of circulation, secretion, innervation, and calorification.

2d. This general disturbance called *fever*, is the result of the action of a poisonous material circulating in the blood, altering its properties, and consequently its relations to all the tissues; and, finally, manifesting a special affinity, first, for the skin, second, for the mucous membrane and glands of the fauces, and third, the kidneys.

3d. The special affinities here mentioned, cause an early lodgement of more or less of the poison in the cutaneous tissue, and the consequent development of specific inflammations in the form of exanthems, vesicles, or pustules. The affinity, especially of the scarlatina and rubeola poisons, for the mucous membrane and glands of the fauces, is scarcely less uniform; and owing to the greater vascularity of the structures, often results in the development of inflammation of far greater intensity and danger than that in the skin. Whether these poisons possess a special affinity for the structure of the kidneys, as indicated in the second proposition, or whether the irritation of those organs, resulting in scantiness of secretion, albuminous urine, and ultimate uremic poisoning or dropsical effusions, is simply the effect of an effort on the part of the secreting structure of the kidneys to eliminate the poison from the blood, we may not be able to determine.

4th. The more complete the lodgement and isolation of these poisons in the cutaneous texture, in the early stage of these diseases, the more mild will be all their subsequent progress. On the other hand, the less perfectly the poison becomes lodged in the skin in any given case, the more persistent will be the general febrile action, the more rapidly will the whole mass of the blood become impaired in its properties, and consequently the greater will be the danger of serious internal complications, or of the supervention of typhoid and malignant symptoms.

If the foregoing general summary is correct, or even proximately so, it affords a basis on which can be founded some important practical maxims for the treatment of this very important class of febrile diseases.

The first and most obvious indication to be fulfilled in the treatment, is the use of such remedies as will directly neutralize the poison in the blood, and thereby obviate most of its effects upon the tissues. Unfortunately, we are not acquainted with any such available antidote. Belladonna, which has been much used both as a proplylactic and remedial agent, probably has no other effect than to lessen the susceptibility of the tissues, and to aid in determining to the cutaneous surface. Chlorine and the chlorates undoubtedly possess properties more nearly akin to antidotes or destroyers of these and other animal poisons.

We have used them for several years, in all stages of eruptive fevers, and generally with benefit.

Failing to neutralize the poison, the next object in the treatment is to mitigate its irritative action on the tissues, and to aid its natural tendency to the skin, either for lodgement or expulsion. For this purpose, the union of anodyne and diaphoretic properties are required, with a careful avoidance of all those active evacuents that would divert the circulation, and consequently the poison, to internal structures, and thereby directly counteract the natural tendency to relief. On this account, active cathartics are particularly objectionable in these fevers.

A third object in the treatment is to counteract local internal inflammation, whether in the throat, kidneys, or mucous surfaces; and to sustain, generally, the vital powers of the patient.

